# Factors influencing recruitment to research: qualitative study of the experiences and perceptions of research teams

**DOI:** 10.1186/1471-2288-14-10

**Published:** 2014-01-23

**Authors:** Lisa Newington, Alison Metcalfe

**Affiliations:** 1 NIHR Biomedical Research Centre, Guy’s and St Thomas’ NHS Foundation Trust and King’s College London, Guy’s Hospital, SE1 9RT, London, UK; 2 Florence Nightingale School of Nursing and Midwifery, King’s College London, James Clark Maxwell Building, SE1 8WA London, UK

**Keywords:** Participant recruitment, Clinical research, Qualitative

## Abstract

**Background:**

Recruiting the required number of participants is vital to the success of clinical research and yet many studies fail to achieve their expected recruitment rate. Increasing research participation is a key agenda within the NHS and elsewhere, but the optimal methods of improving recruitment to clinical research remain elusive. The aim of this study was to identify the factors that researchers perceive as influential in the recruitment of participants to clinically focused research.

**Methods:**

Semi-structured interviews were conducted with 11 individuals from three clinical research teams based in London. Sampling was a combination of convenience and purposive. The interviews were audio recorded, transcribed verbatim and analysed using the framework method to identify key themes.

**Results:**

Four themes were identified as influential to recruitment: infrastructure, nature of the research, recruiter characteristics and participant characteristics. The main reason individuals participate in clinical research was believed to be altruism, while logistical issues were considered important for those who declined. Suggestions to improve recruitment included reducing participant burden, providing support for individuals who do not speak English, and forming collaborations with primary care to improve the identification of, and access to, potentially eligible participants.

**Conclusions:**

Recruiting the target number of research participants was perceived as difficult, especially for clinical trials. New and diverse strategies to ensure that all potentially eligible patients are invited to participate may be beneficial and require further exploration in different settings. Establishing integrated clinical and academic teams with shared responsibilities for recruitment may also facilitate this process. Language barriers and long journey times were considered negative influences to recruitment; although more prominent, these issues are not unique to London and are likely to be important influences in other locations.

## Background

Participant recruitment is vital to the success of a research study, and yet many research projects fail to recruit a sufficient number of participants [1]. Increasing participation in clinical research has become a key area of focus within the NHS, with the aim of facilitating evidence-based policy, improving health outcomes and reducing health inequality [2]. The identification of optimal recruitment methods is gaining interest and a recent systematic review of strategies aimed at improving recruitment to randomised controlled trials (RCTs) identified 45 relevant studies and categorised six types of intervention: trial design, obtaining consent, approach to participants, financial incentives, training for recruiters and trial coordination [3]. Overall, the general strategies found to be effective in improving recruitment included: making telephone reminders to non-responders, having opt-out procedures where potential participants are required to contact the trial team if they do not want to be contacted about a trial, and having open rather than blinded trial designs [3]. It is not known whether more trialists are now adopting these strategies, or if they are proving successful in other settings or for other research methodologies.

Attempts to optimise recruitment and retention for non-interventional research studies include a range of techniques, such as using large sampling frames, sending reminders, running wide-scale publicity campaigns, providing free helplines and providing material in the respondents’ own languages [4]. While there may be universal elements to improving clinical research recruitment, reports of successful recruitment strategies for non-intervention studies are often directed at the particular target demographic group, for example: African American Elders [5], palliative care patients and their carers [6], adolescent mothers [7] and individuals from minority groups [8]. It is clear that recruitment and retention strategies need to be relevant to the target population and the research methodology used, and therefore the optimum strategy is likely to vary. However, further investigation of research recruitment according to different study designs is required to enable an evidence-based approach to recruitment.

The views of the researchers and clinicians involved in participant recruitment are beginning to be explored. We recently conducted a systematic review and thematic meta-synthesis to investigate this subject and found that the recruitment process could be defined by five key themes: building a research community, securing resources, the nature of the research, professional identities, and recruitment strategies [9]. Across all five themes there were reports of competition and compromise. Competition arose over funding, staffing and participants, and between clinical and research responsibilities; whilst compromise was needed to create study designs that were acceptable to patients, clinicians and researchers. Overall the views of researchers and clinicians were similar, which was partly explained by the overlapping elements of their roles.

The factors and situations that prompt some individuals to agree to participate in clinical research when others decline have also received attention, with the hope of informing new recruitment practices. However, to date, this work has been predominantly directed at a single medical condition and there have been varied findings [10-14].

Geographical location has been shown to influence recruitment rates to RCTs, with large cities such as London associated with poorer recruitment [15,16]. Possible suggestions for lower recruitment rates in London are the more varied ethnic population (individuals who are traditionally more difficult to engage in medical research), higher population mobility (individuals potentially missing invitations or reminders to participate), and more university hospitals (creating *research fatigue* as individuals are repeatedly approached to participate in research) [15]. Research teams in London therefore not only have to contend with the recruitment issues faced elsewhere, but may face an additional set of issues associated with their location.

The aim of the current study was to identify and understand the factors affecting recruitment to clinically focused research in London, UK, with the aim of mapping the existing strategies and informing new approaches. This study adds to existing work by exploring pertinent themes that arose across different clinical areas, study designs and researcher roles, providing a broad view of the factors that researchers consider important for the recruitment of clinical research participants. The following questions were explored:

1) What do researchers perceive to be the influential factors in recruiting participants to their clinically focused research?

2) What steps do research teams take to optimise recruitment to their studies?

3) What are researchers’ perceptions of why potential participants consent or decline to participate in their research?

4) Does being located in London create any additional issues with recruitment?

## Methods

A convenience sample of three research leads involved in clinically focused research and based in teaching hospitals in South London were identified and invited to participate in a one-off interview to discuss their experiences and perceptions of recruiting participants for their studies. The phrase *clinically focused research* was defined as any medical research requiring an individual’s consent to participate, including donation of tissue samples, observational studies and RCTs, and the discussion was limited to recruiting adult patients able to give informed consent. The interviews were semi-structured and used non-directive, open-ended questions based on topics identified from preliminary discussions with clinical researchers and from the existing literature; the topic guide is listed in Table 1. Each participant was asked to identify other members of their team with differing roles and responsibilities, and a purposive sample of these individuals was also invited to participate in the study. The same topic guide was used throughout and additional individuals were identified as necessary to ensure a broad mix of research professions were included, and to enable interviewing to continue until saturation was reached. All interviews were conducted face-to-face by the primary author in early 2013 at locations chosen by the participants. The interviews were audio recorded and transcribed verbatim.

**Table 1:** Interview topic guide

**Topic heading**	**Issues discussed**
*Participant characteristics*	Role in research team
	Current projects
	Reasons for working in research
*Strategies and processes of recruitment*	General recruitment strategies
	Strategies for particular demographic groups
	Responsibility of recruiting
*Thoughts on recruitment*	Being a research participant
	Why people agree to participate
	Why people decline
	Increasing participation
	Increasing public awareness of clinical research
*London*	Specific issues with location

### Analysis

The interview data was analysed using the framework method established by Ritchie and Spencer [17]. The framework matrix was developed using NVivo 10 software (QSR) and incorporated the interview topic guide, ideas from the existing literature and prominent themes identified from a preliminary review of the transcripts. The transcripts were coded line by line and additional themes were entered into the matrix where necessary. The matrix was populated with summarised data according to participant and theme, and used to identify common and divergent issues in answer to the study research questions.

### Ethical approval

This study was approved by the King’s College London, College Research Ethics Committee (Reference PNM/12/13-106). All participants gave informed consent to be interviewed. All but one participant also consented to anonymous quotes from their interviews being used in the resulting reports and publications.

## Results

A total of 15 individuals were invited to participate in the study, of which 11 agreed to be interviewed. Participant demographics are shown in Table 2. One speciality registrar declined to be interviewed, citing that his role was predominantly clinical not research-based, and three speciality registrars did not reply to their invitations. The mean interview duration was 28 minutes, ranging from 19 to 48 minutes.

**Table 2:** Participant demographics

**Role**	**Team**	**Male**	**Female**
Consultant, actively involved in clinical research	A, B	2	-
Speciality registrar*, actively involved in clinical research	A, C	1	1
Clinical research scientist	B, C	-	2
Research nurse	A, B, C	1	3
Clinical research associate	A	-	1
**Total**	-	**4**	**7**

*Medical doctor receiving advanced training in a specialist area.

The information provided has been limited to preserve the anonymity of the interviewees and their teams.

Interviewees were involved in a range of studies, all outpatient-based and run as part of three research teams (A, B and C) in three tertiary care hospital sites in South London. Study designs included a first-in-man drug trial, longitudinal observational studies, laboratory studies requiring one-off anonymous tissue samples, genetics studies, trials of therapy interventions, and physiological studies. All research teams carried out research with patients and healthy volunteers, and most interviewees had volunteered themselves as study participants at some stage. With the exception of the two clinical research scientists and the clinical research associate, all participants were also involved in clinical activities as part of their role. When asked why they became involved in clinical research, all participants reported having an interest in research at an earlier point in their career and acting upon this for a variety of reasons including: an extension of a previous role, the desire for more control over their work, part of their current training, to learn more about evidence-based medicine, to do something worthwhile, to improve job satisfaction and to ensure more sociable working hours. All interviewees were educated to degree level, four had gained a PhD and two were working towards a PhD or MD. All participants acknowledged difficultly in recruiting research participants and mentioned particular strategies or modifications that were made to improve recruitment within their teams. The general perception of recruitment was that it is hard to recruit the desired numbers in the allocated time and that more often than not, extensions to the recruitment period are required.

### Influential factors in the recruitment of participants

Numerous factors were identified by the interviewees as influential in the recruitment of research participants and these were categorised into four main themes: infrastructure, nature of the research, recruiter characteristics and participant characteristics.

#### Infrastructure

The need for access to potentially eligible participants was emphasised throughout. Collaboration between hospital clinicians, GPs and researchers were viewed as essential for the identification of eligible patients and to avoid clinician gatekeeping. All research teams had established systems to facilitate the identification of patients, but there was awareness that potentially eligible patients seen in other departments or hospitals were frequently inaccessible.

*“There will be a lot of patients going to [smaller hospitals], who could be enrolled in studies, but they’re not available there. They are available here. If they knew that we were doing it, and there was a mechanism for moving those patients for the duration of the trial here, I would think everyone would be happy. But there isn’t” .* (Consultant, team A)

One team had developed a strategy where local hospitals were encouraged to identify eligible patients and refer them to the participating site for the duration of the trial. Whilst this was seen as a positive step, it was also acknowledged that greater recognition for the referring sites, in terms of funding and co-authorship, would be required to improve uptake.

The preparatory work carried out by research teams was considered highly influential in the success of recruitment. Screening patient records, identifying eligible patients, preparing appropriate recruitment material and ensuring that the relevant clinicians and researchers were fully informed about the study, were all recommended. These tasks were primarily the responsibility of the research nurses and research associates.

*“Here, we do look through the clinic list and, myself on the busiest days, will look at the past three clinic letters and see if they’re going to be suitable, or if they’re already on the study. We do recommend that’s the best way to find patients. And then we’d print the relevant paperwork and put that in the notes, so the doctors can see. So then they don’t even have to think about it, it’s just there. I think that works best. I would say that maybe about half of places do that, because they haven’t got time. They haven’t got time to do the prep” .* (Clinical research associate, team A)

One suggestion to improve access to patients was the use of opt-out systems. This was mentioned with reference to patients being required to opt out of research teams contacting them about relevant research projects, but was also discussed with regard to opting in to the routine donation of anonymous tissue samples (surplus to requirements for clinical tests) for clinically focused research. Neither system was currently in place.

Issue with the regulations surrounding ethical approval and the content of participant information sheets were commonly discussed. The interviewees thought that the approval process was too slow, which created delays in starting recruitment and raised concerns that their departments would get overlooked for involvement in multi-centre studies in favour of sites with faster turnaround times.

*“We certainly need to improve the speed with which we’re able to take a study from application through to actually being run. We are unbelievable slow. Unbelievable top heavy with regulation… It often means, locally, that we get bypassed in these programmes” .* (Consultant, team A)

The interviewees were also concerned that the information required by ethics committees led to the participant information sheets becoming excessively long and detailed, and off-putting to patients. The researchers were aware of the conflict between ensuring patients had sufficient information about a study to make an informed decision about participation, and providing accessible study literature, however many interviewees believed that with the current format, patients did not actually read the information sheets provided, instead relying on verbal discussions to make a decision about participation.

*“I get a few who will [read the patient information sheets], but nobody does. I would say 98% of people don’t read it. I do a summary of what is important to them” .* (Specialty registrar, team C)

Several researchers suggested inviting patients and members of the public to sit on ethics committees to provide feedback on this issue and one research team had implemented a strategy to use more images and pictures in their participant information sheets to improve readability.

Increasing public awareness of clinically focused research was widely thought to have the potential to improve research recruitment, with the exception of one interviewee who felt that people would only be interested in research when their health was affected. Whilst there were many comments on the need to increase awareness of research within hospitals and other healthcare facilities, interviewees had few suggestions of how this could be improved. There was frustration at the lack of media coverage or celebrity endorsement within their clinical areas, compared to the numerous high-profile campaigns for areas such as cancer research. However, the media was viewed as having both positive and negative effects on recruitment, depending on the nature of the coverage.

#### Nature of the research

The influence of the type of research on participant recruitment was discussed by all interviewees. It was noted that clinical trials were harder to recruit for than observational studies because they require greater commitment from the participants in terms of time and risk. The interviewees also acknowledged the difficulty between designing studies that were appealing to potential participants and ensuring they were scientifically robust.

*“We wanted it to be a good trial from the beginning. So it wasn’t just ‘everybody gets [the intervention] and let’s see what happens’. Although that would have been much easier and might have given us the answer quicker. So it’s placebo controlled, randomised, double blind. Not only are we asking these people to possibly risk their lives, but they might not get it anyway” .* (Clinical research scientist, team B)

Some studies incorporated open label or crossover phases after the initial RCT, which was believed to make the study more acceptable to patients. Other recommendations, such as allowing patients to have their study blood tests carried out in the community and offering evening and weekend research appointments, were suggested to reduce the time burden of research participation, but these strategies had not been adopted.

*“I guess the big thing would be to try and reduce the burden of commitment to patients, as much as possible. If there was any chance that they could have research bloods taken with GPs, or in their local community, or research nurses could go and take the blood in their home, to avoid this” .* (Research nurse, team A)

Payment for research participation was also discussed. Research leads highlighted the ethical issues associated with paying patients for research participation, whilst others acknowledged the role of payment as a driver in recruiting people to participate in their work. The semantics of this issue were important, with one interviewee stating that while it was unethical to *pay* patients to participate in research, there was the need to explore *“being able to financially help volunteers better” .* (Consultant, team A).

#### Recruiter characteristics

It was widely reported that patients were more likely to agree to participate research if they were asked by a medical doctor, specifically their usual doctor. Even for observational studies, which do not require a doctor to take consent, it was noted that recruitment was more successful if the doctor mentioned the study to the patient before the research nurse provided a more detailed explanation. In this respect, successful recruitment was seen as a team effort.

*“Our clinicians are so pro-research they are very good at introducing it into the clinical consultation, which really helps, because if it’s first mentioned, I think, by a clinician it’s considered just a normal part of the clinical care, then I think people are sometimes a bit more accepting of it” .* (Research nurse, team A)

In addition to the recruiters’ professional roles, their personality and knowledge of the research project were also considered influential. Although all interviewees had undergone the relevant research and ethics training, none had received specific training in recruitment. There was debate on whether it was possible to teach the *art* of recruitment and if so whether this would be useful. The more experienced researchers felt that specific training was unnecessary as recruitment style and strategy vary depending on the clinical speciality and the particular study involved, and on-the-job experience was believed to be more important that generic recruitment training. It was also suggested that an individual’s personality was central to their recruitment success, an aspect that is difficult to teach.

“*The art of getting people in; it’s not clear. If I couldn’t recruit to trials, I wouldn’t be doing trials… some of my colleagues are good at recruiting, some aren’t quite so good. Trying to tell someone what to do is just not helpful, is it?*” (Consultant, team B)

*“Then it’s also your personality. I think patients, they need to trust you. If you are a little bit unsure about something – not about the protocol itself, because that changes and you can’t expect to know a thousand pages of protocol – but that you are confident. Holding their hands all they way during the study” .* (Research nurse, team B)

The less experienced researchers believed they would have benefited from additional support during the early stages to learn how to optimise their recruitment success, but acknowledged that a general training programme was unlikely to be appropriate for all recruitment situations.

Interviewer: “Did anyone talk to you about recruiting?”

*Respondent: “No, but it would have been helpful… No-one spoke to me and gave me any advice… Although studies are so different and patient groups are so different, that it’s probably slightly different for everyone” .* (Specialty registrar, team A)

The clinical research scientists expressed frustration at being reliant on clinicians to recruit patients for their research, especially as they had completed the prerequisite training and had recruited patients previously; however current regulations prohibit non-clinicians from recruiting patients.

*“I don’t know why they don’t think [scientists] can consent people here. We used to be able to. It’s only the last few years that we’ve not been able to. We’ve done all the consent courses and everything” .* (Clinical research scientist, team B)

#### Participant characteristics

All interviewees thought that certain patients were more likely to agree to participate in clinically focused research than others. The reported reasons for this are explored in more detail in the section “Why do some individuals consent to participate and others decline?”, however it is important to highlight that for a potential participant to either consent or decline to participate in research, they must first be invited. This links to the previous issues of identifying and accessing eligible patients, but also relates to situations where potentially eligible patients may be denied the opportunity to take part. For example, several interviewees mentioned that individuals who do not speak or understand English are unable to participate in the majority of studies due to the absence of funding for interpreter and translation services.

*“…that’s actually something we really need to think of as a team going forward with recruitment, because at the moment we’ve said, for example, if patients come with interpreters or they have no English, then we haven’t included them” .* (Research nurse, team A)

One interviewee recalled using interpretation services in the past, but only as a last resort due to the additional workload created.

*“It did happen in the past, that for some protocol it had been waived that you can have an interpreter, which can’t be a relative. Because it needs to be an independent interpreter. It needs to be really last chance, because it’s a lot of work, extra, on top of what you have to do” .* (Research nurse, team B)

Where potential participants did speak sufficient English to be eligible for participation, but it was not their first language, some interviewees reported lower recruitment rates compared with native English speakers. Suggested reasons for this included communication issues or a general increased reluctance to participate in clinical research.

*“Potentially there have been times in the past where I’ve felt that this person’s not really taking in what I’m saying, for various reasons, whether that’s to do with language differences, English not as a first language” .* (Research nurse, team A)

### Steps taken to optimise recruitment

Table 3 shows the recruitment strategies and specific techniques employed by the research teams and the interviewees’ suggestions of techniques to further improve recruitment. The recruitment strategies were divided into three main themes: preparation and planning, engendering patient support, and collaboration with clinicians. The majority of suggestions to improve recruitment were targeted at making research participation more appealing and less time consuming for patients.

**Table 3:** Steps taken by research teams to optimise recruitment and interviewees’ suggestions for improvement

**Strategy**	**Techniques employed**	**Suggestions for improvement**
*Preparation and planning*	• Assess feasibility before embarking on a study	• Create shared research databases of potentially eligible patients
	• Pre-screen clinic notes to identify potentially eligible patients	• Increase speed of ethical approval process
	• Establish patient identification centres in surrounding hospitals	
	• Ensure research nurses are available to discuss study during patient’s clinic appointment	
*Engendering patient support*	• Research introduced by patient’s doctor	• Increase public awareness of clinical research
	• Advertise the study, but also approach patients individually	• Opt-out systems
	• Explain importance of clinical research for improvements in healthcare	• More accessible participant information sheets
		• Increase use of information technology
	• Discuss the multidisciplinary team involved in research	• Support patients without English as their main language
		• Reduce time commitment required and increase flexibility of appointment times
		• Social events for trial participants
		• Increase financial support for participants
*Collaboration with clinicians*	• Establish integrated clinical and academic teams	• Greater collaboration with primary care clinicians
	• Hold research meetings and provide regular updates and feedback	
	• Enlist a dedicated study co-ordinator	
	• Give prizes for successful recruitment	

### Why do some individuals consent to participate and others decline?

The interviewees believed that the main reason why patients agreed to participate in their research was altruism, including the desire to help future patients and the wish to give something back to the hospital and team that cared for them. For the latter, researchers were clear to point out their duty to ensure that research participation was truly voluntary, rather than an obligation.

*“A common thing tends to be ‘you’ve done so much for me, I’m quite happy to do anything for you’. Which is a sort of double edged sword actually, because that’s very generous of them, but actually you want them to participate because they want to, and you have to say ‘well you don’t have to’, and you’ve got to think that they’ve actually understood” .* (Research nurse, team A)

There was also a general consensus that many individuals who took part in clinically focused research valued the potential benefits of participation, namely the opportunity to access additional health checks, novel treatments, increased contact with clinicians and the clearly defined plan of care. For researchers who provided payment for participation in their studies, financial gain was also viewed as an important motivator.

*“Some of the studies that we run here, we pay £50 a visit. So it’s also to do with people need a bit of extra cash at the moment” .* (Research nurse, team C)

Furthermore, patients who were interested in the research question and believed that clinical research was worthwhile were considered more likely to accept the invitation to participate. As discussed previously, the nature of the research was also viewed as highly influential, with patients preferring to participate in non-interventional studies.

*“I think it’s much easier to recruit for an observational study. Because we’re not doing anything that could harm them” .* (Specialty registrar, team A)

No particular strategies were employed to recruit patients of different ethnicities or socio-demographic backgrounds, with the common belief that recruiters attempt to invite all eligible patients to participate, regardless of their background. Despite the fact that recruitment was limited to English language speakers, most researchers felt that they recruited a good spread of the local population, although this did depend on the clinical area under investigation and the time commitment involved.

*“I suppose retired patients have probably said ‘yes’ more willingly. For our study, we are requiring them to have extra tests. Some of the patients have said they are worried about time. Or getting here from work earlier” .* (Specialty registrar, team A)

For patients who declined to participate in clinical research, the predominant reasons were thought to be practical. Patients who were working were unable to take extra time off work for research appointments and the additional travel required to attend the hospital was also believed to be off-putting, especially for patients who did not live locally.

*“I think for some, mainly it’s time I’d say. Because often they’ve been sat in the waiting room for up to an hour already. So when it gets to the point where they’ve had their appointment, they’ve been seen by a nurse… they’re just like ‘I’ve just not got time’. I think that’s the main issue” .* (Clinical research associate, team A)

Fear was also considered important, mainly with respect to clinical trials. Fear of taking new drugs, fear of additional diagnoses being discovered from extra screening, fear of needles, fear of symptoms worsening and fear of the storage of tissue or genetic information were all suggested. Language was also thought to play a role. As discussed in the section “Participant characteristics”, some interviewees observed that individuals who spoke English as an additional language were more reluctant to participate compared with native English speakers.

*“I have noticed sometimes, I’ve not quantified this yet, because we haven’t analysed out results, but people who maybe don’t have English as a first language are probably a bit more reluctant” .* (Speciality registrar, team A)

### Specific issues for London

When asked specifically about recruitment issues associated with their location in London, the researchers’ responses fell into two main themes: local research community and patient population.

#### Local research community

The interviewees described successful research communities within their own teams, although there was a lack of collaboration with local primary care services. It was suggested that establishing shared research databases and other systems to identify and access potentially eligible patients across different service providers would be beneficial for study recruitment, but that specific initiatives would be needed to facilitate this.

*“It’s hard because, in my view, if you really want to do it, it will cost money. It will involve someone, a GP with a research interest in the catchment area. For example, they call it GPSI, which is a GP with a specialist interest in something, rheumatology or haematology et cetera, but one would have research, just purely doing research” .* (Specialty registrar, team C)

In addition, researchers reported delays in the process of gaining ethical approval for their studies and a lack of financial support for in-house academic research, suggesting that local improvements could be made to these systems.

Despite these recommendations for improvement, the interviewees were generally positive about working in London and the level of research support provided.

*“I think in terms of being in a big London teaching hospital, we are more geared up to research, just from personal experience having worked in district generals in [UK county], there was no set up for research and it was very much a minor thing, and if anyone was doing something, they didn’t have research nurses, it was very much clinician led. It was set up in their own interests really, their own studies. So the fact that we have a forum for research nurses here, and we are trying to actively put out the research message” .* (Research nurse, team A)

#### Patient population

It was noted by the researchers that patients attending hospital appointments in London frequently report long travel times and this was believed to be detrimental to recruitment. This was attributed to the broad catchment area for tertiary healthcare, plus the large number of people who commute into London for work. Interviewees reported difficultly recruiting patients with long journey times, especially if research participation involved additional visits.

*“There’s quite a large population of people that travel in. I guess that will affect people taking part in research. Because if they’re having to travel from Hertfordshire, that’s going to put people off, because yes, you can give them their travel expenses, but you can’t give them their three hours back” .* (Research nurse, team C)

Being a tertiary care centre was also thought to have a positive effect on recruitment, with researchers commenting that patients may be more likely to trust an invitation for research participation from a specialist centre.

*“So people do come in from other hospitals. Again, you have a wider group. Also, they tend to be, in a way, more sick. More likely to listen to the medic who’s telling them, ‘this isn’t a bad thing’” .* (Clinical research scientist, team C)

The interviewees also discussed the diversity of the local population, and as mentioned previously, the lack of interpretation and translation services for research resulting in potentially eligible patients being excluded. However, in general it was felt that the researchers were able to recruit representative samples of their local populations.

Although all these issues were important to researchers, it was also acknowledged that most locations have problems with recruitment and that having sufficient resources and research staff should perhaps be considered more important than the location.

*“I wouldn’t say there are any huge differences that I can think of. I think it really does depend on the staff and the resources that they’ve got, rather than the actual hospital and the patients coming in” .* (Clinical research associate, team A)

## Discussion

The primary aim of this study was to identify the factors that researchers perceive as influential in the recruitment of participants to their clinically focused research. Infrastructure, the nature of the research, recruiter characteristics and participant characteristics were all deemed important. The first three themes are, in theory, more amenable to modification than the last, for example through the development of systems to improve identification and access to eligible participants [18], designing studies with reduced participant burden [10] and ensuring that recruiters have the appropriate knowledge and skills [19]. The discussion of participant characteristics focused on the concept that certain patients were thought more likely to agree to research participation than others. The danger with such an observation is the potential for recruiters to stereotype potential participants based on previous experiences, and therefore choose not to approach individuals who are otherwise eligible. As the NHS constitution pledges to inform all patients of research studies that are relevant to them and in which they may be eligible to participate [20], recruiters must be aware of the potential to deviate from this duty. In reality, the decision to participate in clinically focused research is frequently multifaceted and requires potential participants to consider the personal pros and cons of taking part at any given time [13]. The research nurses interviewed for the current study raised this point and explained their attempts to approach all eligible patients, regardless of any preconceptions about whether or not they would agree to participate.

The general perception that doctors are more successful at recruiting research participants than nurses has been explored previously. Donovan et al. [21] found no significant difference in recruitment rates between urology consultants and nurses for a prostate cancer RCT and calculated that nurses were more cost-effective recruiters, despite spending longer on average with each patient. In the current study, recruitment was viewed more as a team effort. Having the doctor mention research participation as part of the routine consultation was thought to be beneficial, as was having integrated clinical and academic teams on site. However, these strategies require sufficient staffing and resources and rely on specific funding for research posts [9]. The possible recruitment benefits of having an established therapeutic relationship with potential study participants [22], sharing similar cultural backgrounds or languages [23], and employing peer recruiters [24,25] have all been explored in the literature. However, the influence of the recruiter-participant relationship was not widely discussed by the interviewees, nor were the subjects of culture and ethnicity. There was a general consensus that recruiters adopted the same recruitment strategies for all demographic groups, but observational investigations of recruitment practices would be beneficial to further explore these issues. The use of eligibility criteria that include only those who speak sufficient English was attributed to a lack of resources available for interpreter services. Resource limitations would also restrict the use of peer recruitment programmes or other strategies aimed at including minority groups. As recruiting a representative sample is essential for the generalisability of research findings [26], additional investigation of this issue is required.

The research scientists interviewed were disappointed that they were no longer permitted to discuss their study directly with potential participants. This finding echoes the views of biomedical research scientists involved in placental perfusion studies [27]. The scientists raised legitimate concerns that the individuals involved in recruitment did not have sufficient knowledge of the intricacies of the study to be able to fully explain the background and rationale to potential participants, or to answer questions about particular methodologies [19]. It in current study, it was local, rather than national, policy that dictated the exclusion of research scientists from recruitment activities. The potential benefits of allowing research scientists to recruit participants to their research include reducing the workload for clinicians, providing expert knowledge of the study processes and rationale, and separating research recruitment from routine clinical care. The potential drawbacks include the research scientist having a vested interest in the research without the balance of coexisting clinical duties, and the absence of a previous therapeutic relationship with the patient. Further exploration of this issue is required, however it may be advantageous to consider including clinical research scientists as part of the recruitment team, with safeguards to guarantee that patients are not exploited.

The recommendation to use an opt-out system, where patients are required to contact the research team if they do not wish to be invited to participate in clinical research, was made in a recent systematic review [3]. Several interviewees suggested that this might be a beneficial system, however this strategy is not currently employed, and further work is required to pilot the use of *opt-out* within these settings. A variation of this strategy, where patients are invited to opt-in to the anonymous donation of surplus tissue after clinical tests, was also discussed. This type of tissue *biobanking* is available at the interviewees’ hospital sites for patients with a diagnosis of cancer, but is not routinely adopted in other clinical areas. Further research into the extension of biobanks to include other clinical specialties appears warranted [28].

The use of open, rather than blinded trial designs, and telephone reminders were also recommended by Treweek et al. [3]. There is debate over the utility of open study designs due to the potential for increased bias [29], but this methodology is gaining support [30]. The interviewees used modified versions of this strategy, such as having an open or crossover phase after the main trial, and believed this was beneficial for recruitment. None of the interviewees specifically discussed the use of telephone reminders.

The recruitment strategies employed by the interviewees were similar to those identified in our recent meta-synthesis, although there was less focus on emphasising the benefits of research participation in the current study [9]. In addition to the possible coercive aspect of *emphasising the benefits*, the interviewees believed that altruism was the key reason for patients accepting the invitation to participate, and therefore strategies based on highlighting potential personal benefits would not sit with this premise. The dominance of practical issues as proposed reasons for patients declining the invitation to participate in research have been documented elsewhere [9,10].

The key factors associated with conducting clinically focused research in London were language and travel time. Interviewees were unable to offer interpretation services to facilitate discussions about research with patients who did not speak sufficient English. The most recent government data shows that within the associated South London boroughs between 19.6-20.3*%* of residents do not speak English as their primary language, compared with 15.3*%* and 7.1*%* in the next biggest UK cities Birmingham and Leeds, respectively [31]. The range of primary languages spoken is also greater in the interviewees’ regions, with more than 54 different languages, compared to 36 in Birmingham and 29 in Leeds [31]. Traditionally, individuals from ethnic minorities have been considered less likely to participate in clinically focused research, however studies from the USA suggest that this is not the case and recommend that more needs to be done to ensure access to research for minority groups, rather than interventions aimed at increasing willingness [32-34]. Strategies to aid the removal of language barriers identified in the current study would improve access to research and could potentially increase recruitment, however further investigation is required.

The interviewees also observed that patients with long travel times to the hospital were less willing to take part in research. When designing clinically focused studies, it may therefore be useful to explore the interviewees’ suggestions of increasing the use of information technology for data collection and forming collaborations with local healthcare services to minimise participant travel. As the average commuting time is 48*%* longer in London than elsewhere in the country [35] this factor may be less problematic in other locations, however most tertiary and quaternary healthcare services conducting research are likely to experience similar travel issues.

### Strengths and limitations

The current study adds to previous work by providing experiential reports and perceptions from research teams in three different non-cancer outpatient settings, within a specified geographical location. However, as the research teams involved were based in South London, further work is required to ascertain whether these findings translate to other regions, nationally and internationally.

Although interviewing was continued until saturation, the small sample size in the current study means it is not possible to infer any differences between the experiences and opinions of the different professions within the research teams. Furthermore, the current study relied on information collected from semi-structured interviews, and may have been subject to reporter bias. Attempts were made to minimise the degree of bias by selecting independent research teams and interviewing participants individually. Additional explorations of the researchers’ practices that include observation of the recruitment situation would be beneficial, but were beyond the scope of the current study.

## Conclusion

Infrastructure, nature of the research, recruiter characteristics and participant characteristics were all believed to influence the success of recruitment to clinically focused research. Suggestions to improve recruitment included reducing participant burden, providing support for individuals who do not speak English and forming collaborations with primary care to improve identification of, and access to, potentially eligible patients. Despite the focus on London in the current study, the factors identified are not unique to this location and are therefore likely to be representative of other diverse cities within the UK.

## Competing interests

There were no competing interests in the conduct of this study.

## Authors’ contributions

LN and AM made substantial contributions to the design of the study. LN carried out the interviews, conducted the analysis and wrote the final manuscript. AM advised on research conduct from inception to completion, appraised the analysis process and revised the manuscript. Both authors read and approved the final manuscript.

## Pre-publication history

The pre-publication history for this paper can be accessed here:

http://www.biomedcentral.com/1471-2288/14/10/prepub
